# Health effects of electronic cigarette (e‑cigarette) use on organ systems and its implications for public health

**DOI:** 10.1007/s00508-020-01711-z

**Published:** 2020-07-20

**Authors:** Radhika Seiler-Ramadas, Isabell Sandner, Sandra Haider, Igor Grabovac, Thomas Ernst Dorner

**Affiliations:** Department of Social and Preventive Medicine, Centre for Public Health, Medical University of Vienna, Kinderspitalgasse 15/1, 1090 Vienna, Austria

**Keywords:** Nicotine abuse, Smoking cessation, ENDS, E-cigarette policy, E-cigarette flavors

## Abstract

**Background:**

There has been growing concern over the use of electronic cigarettes (e-cigarettes) in recent years. Although advocated as an aid to smoking cessation, there is increasing evidence of harm not just to the respiratory system, but to all other organs in the body. To give a clearer picture on how e‑cigarettes can affect our health, we gathered an overview of the literature on the various health effects of e‑cigarettes and categorized them into how they specifically affect organ systems.

E‑cigarette exposure has produced a range of stress and inflammatory reactions in the pulmonary system, including shortness of breath, coughing, wheezing, bronchial and pulmonary irritations, and impaired pulmonary function. In the oral and gastrointestinal system, gingival inflammation, sore throat, nausea, vomiting, and diarrhea have been reported. Increased tachycardia and blood pressure were reported reactions in the cardiovascular system. In the neurological system headaches, irritability, anxiety, dependence and insomnia were observed. Other effects included ocular irritation, contact dermatitis, acute renal insufficiency, toxicity and potential carcinogenicity. Nevertheless, studies have found improvements in time-based memory and nicotine withdrawal associated with the cessation of conventional cigarette smoking and switching to e‑cigarette use. Also, toxic and carcinogenic metabolites were reportedly lower in e‑cigarette smokers than in conventional cigarette smokers.

**Conclusion:**

A growing number of studies are showing the adverse effects caused by e‑cigarettes on all human organ systems. Further research on the chemical components, the diverse flavors, and the long-term effects on active and passive users are needed to clarify the implications of e‑cigarette use on individual and public health.

## Background

Electronic nicotine delivery systems, known commonly as electronic cigarettes or e‑cigarettes, are hand-held battery-powered devices simulating the structural, behavioral and arguably physiological aspects of traditional smoking [1]. In each of these devices there is a battery, a reservoir containing liquid, and a vaporization chamber with a heating element [2]. The liquid is a solvent comprising propylene glycol, glycerine, one or more flavorings and usually nicotine [2]. When this liquid is heated, the e‑cigarette creates an aerosol of fine particles that is absorbed through the lungs, rapidly travelling through the heart and delivering nicotine to the brain within a  matter of a few seconds [3].

With the evolution of e‑cigarettes into the third and fourth generations as well as the growing prevalence of their use, there has understandably been increasing concern on their health effects [4–6]. This concern is based particularly on inconsistencies between the actual content and labelling of ingredients of the liquids and emissions, the inadequate quality control on safety and contents of the liquids used, as well as an absence of internationally certified manufacturing sites [7]. In addition, the liquids are freely available via the Internet [8]. No firm conclusions have been drawn so far on the safety of e‑cigarettes, but there has been increasing evidence indicating harm [9]. Even deaths associated with or supposedly attributable to e‑cigarette use have been reported [10].

According to Public Health England, e‑cigarettes were reported to be 95% less hazardous than tobacco cigarettes [11]. Apparently switching to vaping (the inhaling of e‑cigarette vapor) has been claimed to be a useful quitting aid for some smokers, and found to be more effective than nicotine replacement therapy, especially when combined with the support of stop smoking services [12, 13]. However, the American Consensus Study Report on the public health consequences of e‑cigarettes stated that in addition to nicotine, most e‑cigarette products contain and emit potentially toxic substances [14]. They also stated conclusive evidence that exposure to nicotine from e‑cigarettes is highly variable and depends on the characteristics of the device and liquid, as well as how the device is operated; however, toxicants, carcinogens and ultrafine particles known to cause adverse health effects have also been found in them, questioning the legitimacy of their use as replacements [5]. Furthermore, short-term e‑cigarette use was found to have immediate adverse physiological effects similar to those seen with tobacco smoking [8, 15]. Passive exposure to e‑cigarette vapor likewise has the potential to lead to adverse health effects [16].

E‑cigarettes were commercially available in Europe and in the USA as of 2006, with reported use increasing dramatically over recent years; nevertheless, global use seems to remain low [17]. E‑cigarette use among youth has increased in popularity as young people are attracted by the novelty of the device and the added notion that it is harmless or less harmful than conventional cigarettes [3]. Yet, it has become particularly concerning that an increasing number of young people without prior smoking experience are experimenting and initiating with vaping flavors as well as nicotine [2, 18, 19]. This is problematic, because youth are more susceptible to nicotine dependence than adults, and nicotine has adverse effects on brain development [3]. It may be that children and young adults are using e‑cigarettes as a safer alternative to experiment without intending to replace traditional cigarettes [20]. However, it has been reported that adolescents and young adults who smoke e‑cigarettes are much more likely to progress to conventional cigarettes than those who had not smoked e‑cigarettes [5, 21].

The use of e‑cigarettes is also increasing among adults [20]. Prevalence of e‑cigarette use is highest among the unemployed and manual workers, while men are more prone to use it than women [22]. Daily users are more likely to be former cigarette smokers compared to non-daily users of e‑cigarettes [23]. Adults have reportedly been using e‑cigarettes as cessation tools or safer alternatives to traditional cigarettes [20]. Nonetheless, on closer examination this may be more complex. In a review comparing e‑cigarette users, non-smokers were reported to prefer no nicotine to low-nicotine, while smokers preferred high-nicotine e‑cigarettes [19]. Further, unmonitored e‑cigarette use was found to be associated with significantly less quitting among smokers, and most adult e‑cigarette smokers continue to additionally smoke conventional cigarettes [3, 24]. To add to the conundrum, misconceptions about e‑cigarettes are common among pregnant women because of the perception that they are less harmful than traditional cigarettes [25]. Women have been reported to prefer sweet-flavored e‑cigarettes as they were assumed to be less harmful than tobacco flavored ones. Yet, several studies have stated that some sweet and flavored chemicals could be of toxicological concern [19]. Clearly, the potentially harmful effects of e‑cigarettes are being underestimated, summoning the need to screen women in prenatal care for e‑cigarette use, and to convey current information regarding the adverse effects of e‑cigarettes [25].

To give a clearer picture on how e‑cigarettes can affect our health, we gathered an overview of growing evidence in the literature on the various health effects of e‑cigarettes and categorized them into how they specifically affect organ systems. PubMed and Google Scholar were selected as primary databases, from which the search terms electronic cigarettes, electronic nicotine delivery systems, health effects, review and public health were entered. Searches were limited to literature in the English language and those that were published from January 2015 to November 2019.

## Effects on organ systems associated with e‑cigarette use

Table 1 summarizes the adverse health effects in association with e‑cigarette use. Further details are described in the sections that follow.

**Table 1:** Adverse health effects in different organ systems in association with electronic cigarette (e-cigarette) use

Organ System	Symptoms/Diseases	Reference
Respiratory system	*Dyspnea (shortness of breath)* Reaction to components released in aerosol	[9, 26, 27]
	*Cough, wheezing* More often experienced by dual users (e-cigarette + conventional cigarette) than e‑cigarette-only users. Reaction to components released in aerosol	[15, 26–28]
	*Lung, nasal and paranasal cancer* Can be induced when exposed to high doses of released trace metals in aerosol	[27]
	*Chest pain* Common symptom associated with respiratory problems	[26]
	*Hemoptysis (coughing blood)* Diffuse alveolar hemorrhage has been observed	[26]
	*Low oxygen saturation (oxygen saturation of 95% and less)* Among patients breathing ambient air	[26]
	*Tachypnea (rapid breathing)* Breathing rate more than 20 breaths/min	[26]
	*Increased flow respiratory resistance and airway resistance* Flow respiratory resistance was measured at 5 Hz, 10 Hz, and 20 Hz via impulse oscillometry after 5min of e‑cigarette use	[9, 15]
	*Abnormal chest radiograph or computed tomography (CT) including infiltrates, opacities, pneumomediastinum, pleural effusion and pneumothorax*	[26]
	*Induces airway and ocular irritation and pulmonary obstruction* Caused by main component (propylene glycol) as well as trace metals released in aerosol	[9]
	*Irreversible lung injury* Some patients do not recover with medical treatment and vaping cessation	[20, 29]
	*Mild, nonspecific inflammation, acute diffuse alveolar damage, foamy macrophages as well as interstitial and peribronchiolar granulomatous pneumonitis.* Use of systemic glucocorticoids brought about respiratory improvement in the majority of cases	[26]
Gastrointestinal system	*Dry or sore mouth, tongue or throat* More often experienced by dual users (e-cigarette + conventional cigarette) than e‑cigarette-only users	[28]
	*Nausea, vomiting, diarrhea, abdominal pain* Common symptoms	[18, 26]
Cardiovascular system	*Short-term increase in systolic blood pressure* Limited evidence	[14]
	*Increased diastolic blood pressure* Moderate evidence	[14]
	*Tachycardia (increased heart rate) and palpitations* Substantial evidence; more often experienced by dual users (e-cigarette + conventional cigarette) than e‑cigarette-only users	[14, 26, 28]
	*Acute endothelial cell dysfunction, oxidative stress* Substantial evidence	[14]
Neurological system	*Dependency* Substantial evidence with moderate evidence that risk and severity of dependency (depending on product characteristics) may be lower compared to conventional cigarettes	[14]
	*Light-headedness*	[18]
	*Dizziness* Experienced by significantly more dual users (e-cigarette + conventional cigarette) than e‑cigarette-only users	[18, 28]
	*Jitteriness, anxiety, irritability, headache* These symptoms have been reported among adolescents within hours after use	[18, 30]
	*Relaxation*	[18]
	*Lack of concentration and sleeping difficulties* Major source of interference with school and sports participation among youth	[30]
	Tenseness, excitement	[18]
Urogenital system	*Acute renal insufficiency* Resolved with intravenous hydration	[26]
Dermatology	*Contact dermatitis* Chronic dermal exposure to nickel contained in aerosol. Allergic skin reactions and eye irritations may be due to propylene glycol	[27, 31]
Haematopoietic system	*Leukocytosis* More than 11,000 white blood cells/mm^3^	[26]
	*Elevated erythrocyte sedimentation rate* More than 30 mm/h. *Mildly elevated serum aminotransferase* *Mild hyponatremia (low sodium), mild hypokalemia (low potassium)* Reported in approx. one third of the 53 cases	[26]
Not attributable to a specific organ system	*Fever, subjective heat sensations, chills, weight loss, fatigue or malaise*	[26]

### Pulmonary system

E‑cigarette exposure produces a range of stress and inflammatory reactions in the areas of the body in which it comes in first line of contact: the mouth, the nasal passage, the trachea, the bronchial system, and the lungs. Exposure to the main component of e‑cigarette liquid, propylene glycol, has incited airway irritation (similar to ocular irritation) airway obstruction and an increased severity of dyspnea among individuals who did not previously have the condition [9, 26]. Depending on the level of exposure, the trace metals nickel, chromium, cadmium, copper and manganese released from e‑vapors have been reported to induce or have the potential to induce several adverse effects. These include shortness of breath, coughing and wheezing, bronchial and pulmonary irritations, irritations of the mucous membranes in the eyes and upper respiratory tract, impaired pulmonary function, as well as lung, nasal and paranasal cancers [27].

E‑cigarette use has also been reported to potentially induce chronic obstructive pulmonary disease [9]. Effects similar to those seen with tobacco smoking, such as increased airway resistance, decrease in specific airway conductance as well as increase in impedance and overall peripheral airway resistance have been reported [9]. Several case reports have documented adverse effects of e‑cigarette components on the respiratory system [20]. Although cases have recovered with medical treatment and vaping cessation, lung injury linked to vaping may not always be reversible [20, 29]. For instance, a case of an acute onset of respiratory symptoms associated with severe fixed airway obstruction was reported in a 45-year-old man who stopped smoking conventional cigarettes and began high-dose vaping 9 months before the incident occurred [29]. His symptoms and severe obstructive lung disease persisted despite vaping cessation and aggressive medical treatment. Another study reported a case series of eight patients who sustained vaping-associated acute lung injury. While most recovered with corticosteroid therapy, one died [32].

### Oral system

There are studies on the oral health effects of e‑cigarettes suggesting that e‑cigarette aerosols can cause harm to oral health by inducing gingival inflammation: however, there is limited evidence to show that switching from conventional cigarettes to e‑cigarettes improves periodontal disease in smokers [14].

### Gastrointestinal system

The most common gastrointestinal symptoms associated with vaping were epigastric pain, nausea and vomiting, followed by diarrhea and hemorrhage, which Gaur et al. attributed to the trace metals copper and chromium in the aerosol if exposed to high levels [26, 27]. Further reports have documented relapsed ulcerative colitis in two unrelated cases of heavy cigarette smokers after having stopped cigarette smoking and starting e‑cigarette use [33]. In these cases, either the continued use of e‑cigarettes or switching back to cigarette smoking helped alleviate the symptoms. In another case report of a pregnancy health effect linked to e‑cigarette use, a 1-day-old infant suffered gastrointestinal bleeding with abdominal distension and respiratory distress [33]. The infant recovered at 6 months, after having undergone double barrel ileostomy and subsequent surgical procedures. At 9 months, the child’s developmental milestone was met.

### Cardiovascular system

An increase in heart rate and blood pressure due to e‑cigarette use has been reported [9, 20]; however, there has been insufficient evidence associating e‑cigarettes with long-term changes in heart rate, blood pressure as well as cardiac geometry and function [14]. Studies have shown that e‑cigarette use has no short-term effects on cardiac function, and that nicotine does not impair the cerebral pressure-flow relationship [9]; however, there has been one case report of an acute myocardial infarction confirmed by angiography in a previously healthy 24-year-old male after having switched from cigarette smoking to nicotine vaping. He was administered thrombolytic therapy, prescribed medications, and was found to be free of subjective symptoms after 1 month of follow-up [33]. Carbonyls and acrolein found in appreciable levels in e‑cigarettes have been found to exert significant cardiovascular toxicity, while nicotine increased the risk of cardiac arrhythmia [34].

### Cognitive and neurological functions

Studies have found improvements in time-based memory and nicotine withdrawal associated with the cessation of conventional cigarette smoking and switching to e‑cigarette use [9]. It was unclear what kinds of e‑cigarettes were used in these switches. Chronic exposure to nicotine especially in adolescence has been shown to lead to negative long-term impacts on memory and attention [30]. A case of a 7-day history of headaches and 2 seizures was reported in a previously healthy 39-year-old male. This patient’s headaches were resolved on the third day after taking his prescribed medication and abstaining from e‑cigarette use, although he continued smoking 10–15 cigarettes a day with a nicotine patch [33]. A case of acute confusion, somnolence and agitation, along with palpitation and vomiting, was reported in a healthy 24-year-old man who ingested two drops of e‑cigarette fluid [35].

### Carcinogenicity/toxicity/injuries

Although toxic and carcinogenic metabolites were reportedly lower in e‑cigarette smokers than in conventional cigarette smokers, a few e‑cigarette users were found to have higher than expected levels of urinary metabolites of lung carcinogen and tobacco-specific nitrosamine [9]. While there is no available evidence that e‑cigarette use is associated with intermediate cancer end-points, there is substantial evidence that some chemicals present in e‑cigarette aerosols, such as formaldehyde and acrolein as well as the trace metals nickel, lead and cadmium are capable of causing DNA damage and mutagenesis, with potential carcinogenic effects [20, 27]. Additionally, it has been reported that pulegone, a potential human carcinogen, which has been banned by the U.S. Food and Drugs Administration (FDA) as a food additive, is still found in substantial amounts in mint-flavored and menthol-flavored e‑cigarettes and smokeless tobacco products [36].

There have been cases of nicotine poisoning in children and an infant through accidental ingestion of the e‑cigarette refill fluids. These children experienced sudden onsets of vomiting, and in one case tachycardia, grunting respiration and truncal ataxia were reported [33]. All patients’ symptoms resolved with medical treatment; however, there is conclusive evidence that intentional or accidental exposure to e‑liquids (from drinking, eye contact or dermal contact) can result in adverse health effects such as seizures, anoxic brain injury, and lactic acidosis. Also, intentional or unintentional drinking or injection of e‑liquids can be fatal [14]. Chronic dermal exposure from vaping can occur around the perioral area and could potentially result in contact dermatitis and ocular irritation from the nickel and propylene glycol contained in e‑cigarettes [16, 27].

## Discussion and public health implications

From the gathered evidence, e‑cigarette exposure has produced a range of adverse reactions affecting all of the major organ systems. Most of them have been associated with the respiratory system, where a range of adverse effects from throat and mouth irritations to stress and inflammatory reactions, severe respiratory obstruction and even death has occurred. In the gastrointestinal system, e‑cigarette aerosols have been found to cause gingival inflammation and periodontal diseases, nausea, vomiting, diarrhea and abdominal pain. In the cardiovascular system, increased heart rate and blood pressure has been frequently reported, although more serious cardiovascular conditions were rare. E‑cigarette use has also been found to create headaches, jitteriness, anxiety and irritability, and the nicotine variants may have long-term negative effects on memory function. E‑cigarette liquids and aerosols have been found to be skin irritants, have toxic components that are poisonous when accidentally ingested, or may have DNA damaging and carcinogenic effects in concentrated doses.

Despite the growing evidence there are limitations to consider. Firstly, much of the data come from case reports or cases series. Although some of the known toxic effects of the constituents found in e‑cigarettes were evident in biological models, they may not have the exact effects on humans [9]. In some studies, there were large variations in the duration of exposure to smoking or vaping among the volunteers, but in others this aspect was not clearly stated. This makes it difficult to give robust estimates of long-term effects. There is an absence of broader evidence from larger samples of individuals as well as a lack of longitudinal studies that may provide more proof to strengthen the conclusions on e‑cigarette health effects.

In the European Union (EU), there have been substantial differences between the laws, regulations and administrative provisions on the manufacture, presentation and sale of e‑cigarettes in member countries since 2014. Furthermore, there is no EU-wide regulatory framework guaranteeing their safety and quality [37]. The recently revised Tobacco Products Directive only states that manufacturers and importers are obliged to notify national authorities of any product they wish to place on the market [38]. These products need to come with specific warnings, a maximum nicotine content of 20 mg/ml, and a leaflet requirement. In addition, there is a separate prohibition on advertising and sponsorship, as well as annual reporting obligations; however, it also states that as e‑cigarettes display different objective characteristics from those of tobacco products, they are submitted to a more lenient legal regime than that applicable to tobacco products [38].

Likewise, the FDA [39] has requested that establishments selling e‑cigarettes be registered, and that their list of products including labelling and advertisements, tobacco health documents and ingredient listing be submitted, in an effort to increase product awareness and develop future regulations regarding e‑cigarette manufacturing and marketing. Furthermore, a warning statement on packages and advertising for e‑cigarettes is mandatory [39]. Essentially, the regulation of e‑cigarettes is comparable to that of tobacco products in terms of sales to minors and allowable marketing techniques [20].

E‑cigarettes are similarly regulated in 68 countries using existing tobacco legislation, new policies or amendments to legislations. But this could be challenging, as e‑cigarette manufacturers are using sales and marketing tactics along with the far-reaching potential of the Internet to stay ahead of these regulations [40]. On the other hand, around 40 countries including Australia, Singapore, Mexico, Brazil, Argentina and Colombia, have opted to take a different stance and have completely banned e‑cigarettes [17, 20, 41]. Some of the reasons countries have prohibited e‑cigarettes were reportedly due to unintended consequences associated with its use, such as inducing or maintaining nicotine addiction, as well as undermining indoor smoking restrictions or smoke-free air policies [42]. Another perhaps more discerning reason, was applying the precautionary principle in the interests of public health and life until the many questions on the ingredients, effects, and impact on health could be concretely answered [41]. On the other hand, the countries that promote e‑cigarettes for smoking cessation have very little evidence to support their success [43].

While e‑cigarettes have been reported to have the potential to reduce harm caused by conventional cigarettes with users being mainly cigarette smokers wanting to quit, a recent meta-analysis concluded that e‑cigarettes reduced the probability of quitting [24, 40, 44, 45]. This analysis, which mainly included observational studies, highlighted the possibility that unlike in clinical trials, participants in the studies chose rather than were assigned to use e‑cigarettes. One factor that may influence choice is the continued self-administration of nicotine in situations or places where smoking is prohibited [24]. If dual use (concomitant use of e‑cigarette and conventional cigarettes) is common among smokers who take this route to quit, the effect of e‑cigarettes on smoking cessation is doubtful [45]. Furthermore, if people who start using e‑cigarettes to quit smoking are likely to continue using e‑cigarettes even without conventional cigarettes, it is unlikely that transferring this addiction pattern is a viable public health solution, considering the gathered evidence of the risks associated with e‑cigarette use.

Similarly, it is problematic that the popularity of e‑cigarettes among youth and adult non-smokers is increasing through aggressive marketing, attractive flavorings and perceptions of reduced risk. Since they have been reported to serve as a gateway to conventional cigarette smoking, e‑cigarettes can hardly be a tool to improve public health value [7, 45].

According to a report from the European Regulatory Science on Tobacco (EUREST) consortium, the wide variety of chemical components and flavor additives warrant further investigation [7]. It asserts that there are risks of e‑cigarette use associated with design and product flaws as well as inadequate or misleading information with regards to product constituents and industry claims. It further highlights the dangers associated with the possibility of modifying or blending refill liquids and using incompatible devices, resulting in the production of harmful compounds or an alternative consumption of illegal substances.

## Conclusion

Based on the existing evidence, no firm conclusion as yet can be drawn regarding the safety of electronic cigarette (e‑cigarette) use, but a growing number of studies are showing the adverse effects it causes in the respiratory system, the gastrointestinal system, the cardiovascular system, the neurological and cognitive system, the urogenital system, the hematopoietic system, and on the skin. As described in the background, e‑cigarettes have been improving smoking cessation. Nonetheless the evidence is scarce, and as the health effects of continued e‑cigarette use are unknown and underresearched, e‑cigarettes should only be used under close medical supervision. Yet, as e‑cigarettes are relatively novel, sensational and freely available on the Internet, the likelihood of young people starting with them and then moving on to combustible cigarettes is high. E‑cigarettes are regulated to various extents around the world, but their safety and quality is not guaranteed. Given the gathered evidence, the use of e‑cigarettes as a public health tool is questionable.
